# Enhanced recovery after surgery (ERAS) in radical cystectomy patients: from consensus to evidences

**DOI:** 10.1590/S1677-5538.IBJU.2019.04.02

**Published:** 2019-09-02

**Authors:** Marco Moschini, Armando Stabile, Agostino Mattei, Francesco Montorsi, Xavier Cathelineau, Rafael Sanchez-Salas

**Affiliations:** 1 Department of Urology, L’Institut Mutualiste Montsouris, Université Paris Descartes, Paris, France;; 2 Department of Urology, Luzerner Kantonsspital, Spitalstrasse, 6000 Luzern, Switzerland;; 3 Department of Urology, Urological Research Institute,Italy; San Raffaele Scientific Institute, Milan, Italy

Bladder cancer (BCa) is the second most common genitourinary malignancy with 81,190 estimated new diagnoses for 2018 in the United States alone (1). Radical cystectomy (RC) with bilateral pelvic lymph node dissection (PLND) and perioperative chemotherapy is the standard treatment recurrent high risk non-muscle invasive and for muscle invasive BCa (2). However, RC as well as perioperative chemotherapy represent a complex procedure associated with high perioperative morbidity and mortality as a consequence also of the characteristics of the population which is generally affected by multiple comorbidities when compared to other surgical procedures (3, 4). One of the strategies proposed to reduce the perioperative morbidity and mortality consist in the application of an Enhanced recovery after surgery (ERAS) program (5). It consists in a multidisciplinary, multi-element care pathway that aim to standardize and improve perioperative management. ERAS program was firstly introduced in colorectal surgery, where ERAS or fast track surgery pathways have been developed to accelerate recovery by attenuating the stress response. Thereafter, a metanalyses have provided level 1 evidence reporting a reduction of complications, and hospital stay for colorectal surgery patients where ERAS was applied (6, 7).

ERAS protocol has been also proposed in the field of RC, however at the time only limited evidences exist (8, 9). It is an approach comprehensive of preoperative, perioperative and postoperative interventions. During the consensus (5) 34 points were individuated. A preoperative counselling and education with verbal and written information regarding surgery, urinary diversion and planned early recovery is necessary to involve the patient in the therapeutic management. In this period several assessments have to be proposed: a preoperative medical optimization, nutritional assessment, visit by a stoma nurse with advice on stoma and neobladder care, cardiopulmonary exercise testing if indicated, advice and support for cessation of smoking and social issue addressed and discharge planning. Smoke cessation in this setting has been demonstrated to be highly effective in increasing survival and perioperative outcomes after RC (10). These elements required a multidisciplinary team composed by urologists, nurses, nutritionists and social workers.

The day before RC, a carbohydrate loading is required, while no bowel preparation is recommended. The day of RC, solids up to 6 hours and clear fluids up to 2 hours preoperative including carbohydrate loading are permitted. From the anesthetic point of view, no long acting sedatives and a thrombosis prophylaxis with compression stocking and low molecular weight heparin is recommended. Only a limited antimicrobial prophylaxis and skin preparation with clorexidine-alcool. A minimally invasive approach with robotic assisted radical cystectomy is recommended. Fluid restriction and prevention of hypothermia with removal of nasogastric tube in recovery. In the day 2-4 after cystectomy, prevention of postoperative nausea and vomiting, use of chewing gum and unrestricted diet. Drain fluid routinely sent for creatinine at day 2 and drain removed if drain fluid indicates serum creatinine levels. Thrombosis prophylaxis with compression stockings and low molecular weight heparin. Early mobilization, daily nutritional supplements with nutrition goal of 900 kcal/d, fluid/electrolyte, encourage self-care. At day 4, discharge at home is possible when criteria met: pain adequately controlled, independently mobile, regular diet/normal bowel function, component with neobladder or stoma care. These elements seem particularly difficult to be applied in real life setting where the type of health care system are very different across the countries. In fact, a discharge at day 4 seem feasible only in the presence of sub-acute facilities care or dedicated nurse that might help the patients to return to the normal life. At 10 postoperative day is programmed a postoperative visit with the removal of stent 10 without a stentogram and the removal of clips.

The proposal of an optimal international accepted pre, intra and postoperative management of RC patients is an important guide for all the urologists around the world. However, some criticism must be highlighted. First, the number of elements that has to be considered for defining an ERAS protocol is arbitrary. The definition of ERAS itself might be tricky with several articles in the literature which proposed different ERAS approaches comprehensive by different elements and therefore it has really to be questioned if it possible to test an approach comprehensive by so different numbers of elements. A consensus has been developed by world recognized urologists but it remains an expert opinion and therefore a low level of evidence (5), therefore there might be other elements non-included in it or centers claiming to do ERAS but not respecting all the points suggested. In this context it is difficult to understand the differential impact of every point in reducing perioperative morbidity and mortality. Second, not all the elements included in the RC ERAS have the same level of evidences. For example, in contemporary patients, the use of the robotic approach has rapidly increased, surpassing in selected American and European centers the traditional open approach (11). However, the use of robot instead the classic open surgery seem not have (12) enough evidences to be included in the ERAS protocol and its presence in the ERAS protocol seem not justified.

In this sense, ERAS protocol seems an important way to standardize the management of RC candidates. However, there is a need to evaluate in detail every single element included in this protocol before accepting it in the normal routine. The more elements from the ERAS proposal one takes, the better the outcome should be. In the future a standardized ERAS basic approach should be clearly defined and then the intervention is to be particularized by each institution and each patient. ERAS should be evaluated and eventually adopted by each Institution on the basis of their own resources, needs and cultural differences.

## References

[1] 1. Siegel RL, Miller KD, Jemal A. Cancer statistics, 2018. CA Cancer J Clin. 2018;68:7-30. 10.3322/caac.21442 29313949

[2] 2. Alfred Witjes J, Lebret T, Compérat EM, Cowan NC, De Santis M, Bruins HM, et al. Updated 2016 EAU Guidelines on Muscle-invasive and Metastatic Bladder Cancer. Eur Urol. 2017;71:462-75. 10.1016/j.eururo.2016.06.020 27375033

[3] 3. Moschini M, Simone G, Stenzl A, Gill IS, Catto J. Critical Review of Outcomes from Radical Cystectomy: Can Complications from Radical Cystectomy Be Reduced by Surgical Volume and Robotic Surgery? Eur Urol Focus. 2016;2:19-29. 10.1016/j.euf.2016.03.001 28723446

[4] 4. Di Trapani E, Sanchez-Salas R, Gandaglia G, Rocchini L, Moschini M, Lizee D, et al. A nomogram predicting the cancer-specific mortality in patients eligible for radical cystectomy evaluating clinical data and neoadjuvant cisplatinum-based chemotherapy. World J Urol. 2016;34:207-13. 10.1007/s00345-015-1640-2 26198750

[5] 5. Collins JW, Patel H, Adding C, Annerstedt M, Dasgupta P, Khan SM, et al. Enhanced Recovery After Robot-assisted Radical Cystectomy: EAU Robotic Urology Section Scientific Working Group Consensus View. Eur Urol. 2016;70:649-60. 10.1016/j.eururo.2016.05.020 27234997

[6] 6. Gatt M, Khan S, MacFie J. In response to: Varadhan KK, Neal KR, Dejong CH, Fearon KC, Ljungqvist O, Lobo DN. The enhanced recovery after surgery (ERAS) pathway for patients undergoing major elective open colorectal surgery: a meta-analysis of randomized controlled trials. Clin Nutr 29 (2010) 434-440. Clin Nutr. 2010;29:689-90; author reply 691-2. 10.1016/j.clnu.2010.01.004 20116145

[7] 7. Lemanu DP, Singh PP, Stowers MD, Hill AG. A systematic review to assess cost effectiveness of enhanced recovery after surgery programmes in colorectal surgery. Colorectal Dis. 2014;16:338-46. 10.1111/codi.12505 24283942

[8] 8. Azhar RA, Bochner B, Catto J, Goh AC, Kelly J, Patel HD, Pruthi RS, Thalmann GN, Desai M. Enhanced Recovery after Urological Surgery: A Contemporary Systematic Review of Outcomes, Key Elements, and Research Needs. Eur Urol. 2016;70:176-87. 10.1016/j.eururo.2016.02.051 PMC5514421 26970912

[9] 9. Pang KH, Groves R, Venugopal S, Noon AP, Catto JWF. Prospective Implementation of Enhanced Recovery After Surgery Protocols to Radical Cystectomy. Eur Urol. 2017;S0302-2838(17)30660-7. 10.1016/j.eururo.2017.07.031 28801130

[10] 10. Crivelli JJ, Xylinas E, Kluth LA, Rieken M, Rink M, Shariat SF. Effect of smoking on outcomes of urothelial carcinoma: a systematic review of the literature. Eur Urol. 2014;65:742-54. 10.1016/j.eururo.2013.06.010 23810104

[11] 11. Zamboni S, Soria F, Mathieu R, Xylinas E, Abufaraj M, D Andrea D, et al. Differences in trends in the use of robot-assisted and open radical cystectomy and changes over time in peri-operative outcomes among selected centres in North America and Europe: an international multicentre collaboration. BJU Int. 2019;4. [Epub ahead of print] 10.1111/bju.14791 31055865

[12] 12. Rai BP, Bondad J, Vasdev N, Adshead J, Lane T, Ahmed K, et al. Robotic versus open radical cystectomy for bladder cancer in adults. Cochrane Database Syst Rev. 2019;4:CD011903. 10.1002/14651858.CD011903.pub2 PMC6479207 31016718

